# One-Year Outcome of Everolimus-Eluting Stents Versus Biolimus-Eluting Stents in Patients Undergoing Percutaneous Coronary Intervention

**Published:** 2016-04-13

**Authors:** Mohammad Alidoosti, Vahideh Sharifnia, Seyed Ebrahim Kassaian, Alimohammad Hajizeinali, Hamidreza Poorhosseini, Mojtaba Salarifar, Younes Nozari, Elham Hakki Kazazi

**Affiliations:** *Tehran Heart Center, Tehran University of Medical Sciences, Tehran, Iran.*

**Keywords:** *Biolimus-A9*, *Everolimus*, *Stents*, *Drug-eluting stents*, *Outcome assessment*

## Abstract

**Background: **The biolimus-eluting stent (BES), with a biodegradable polymer, has not been previously compared with the everolimus-eluting stent (EES), as a second-generation drug-eluting stent (DES).We sought to compare the 1-year outcome between the PROMUS^™^ stent (EES type) and the BioMatrix^™^ stent (BES type).

**Methods: **From March 2008 to September 2011, all patients treated with the PROMUS™ stent or the BioMatrix™ stent for coronary artery stenosis at Tehran Heart Center were enrolled. The primary end points were 1-year adverse events, comprising death, myocardial infarction, target vessel revascularization, and target lesion revascularization. The secondary end point was stent thrombosis. The Cox proportional hazard model was used to assess the adjusted association between the stent type and the follow-up outcome.

**Results: **From 949 patients (66.3% male, mean age =59.48 ± 10.46 y) with 1,018 treated lesions, 591 patients (630 lesions, 65.1% male, mean age = 59.24 ± 10.23 y) received the PROMUS^™^ stent and 358 patients (388 lesions, 68.2% male, mean age = 59.88 ± 10.83 y) were treated with the BioMatrix^™ ^stent. Before adjustment, the rate of the primary end points was 3.2% and 3.4% in the EES and BES, respectively (p value = 0.925, HR _(EES to BES)_ = 1.035, 95% CI: 0.50 to 2.13). The rate of stent thrombosis was 2% and 1.7% in the EES and BES, respectively (p value = 0.698). After adjustment on confounder variables, there was no statistically significant difference in major adverse cardiac events between the PROMUS^™^ stent and the BioMatrix^™^ stent (p value = 0.598, HR _(EES to BES)_ = 0.817, 95% CI: 0.39 to 1.73).

**Conclusion: **At 1 year’s follow-up, the BES and EES showed similar safety and efficacy rates in the patients undergoing percutaneous coronary intervention with a relatively low rate of adverse events in the 2 groups.

## Introduction

First-generation drug-eluting stents (DESs) were introduced primarily with the goal of reducing neointimal hyperplasia and the subsequent in-stent restenosis. A large number of studies have shown that first-generation DESs reduce the need for target lesion revascularization by 40%–70% compared with the bare metal stent (BMS).^1^^-^^4^ As a potential step forward in stent technology, second-generation DESs were developed with thinner stent struts and more desirable flexibility, deliverability, and polymer biocompatibility.^5^^, ^^6^ Although first- and second-generation DESs with durable polymer have proved to be a successful method for drug loading and drug release,^7^ inflammation and hypersensitivity reaction, induced by the polymer component of these stents, have prompted researchers to develop biodegradable polymer stents in order to overcome this limitation.^8^^-^^11^

In the majority of the previous studies, first-generation DESs and more specifically, the CHYPHER^®^ stent, are considered a gold standard, against which newer stents are compared. Furthermore, novel DESs compared to the first-generation sirolimus-eluting stents (SESs) have shown similar results.^6^ The biolimus-eluting stent (BES), with a biodegradable polymer, has not been previously compared with the everolimus-eluting stent (EES), as a second-generation DES. Additionally, everolimus is an analogue of sirolimus. The present study was, therefore, designed to compare the 1-year clinical outcome of percutaneous coronary intervention (PCI) between patients treated with the BES and those treated with the EES.

## Methods

In a historical cohort design, from March 2008 to September 2011, all the patients treated with the PROMUS™ stent (EES, Abbott Vascular, USA) or the BioMatrix™ stent (BES, Biosensors International, Switzerland) for coronary artery stenosis at Tehran Heart Center were enrolled in this study and were followed up for 12 months. Data on these patients were extracted from a computerized database of prospectively recorded clinical and procedural information on standardized forms during the in-hospital period and at follow-up. Patients with various types of stents in the same or other vessels and those who had not continued dual antiplatelet therapy for up to 1 year were excluded. All the patients provided consent for the use of their records for research purposes, and the study protocol was approved by the local ethics committee. 

PCI was performed by using standard interventional techniques. Before the index procedure, all the patients received ≥ 75 mg of aspirin and 300–600 mg of oral clopidogrel (75 mg once daily at least 3–5 days before the procedure or a 300–600 mg of oral loading dose before PCI). During the procedure, 7500–10000 of IU intravenous heparin boluses were administered, and glycoprotein IIb/IIIa inhibitor was used at the discretion of the physician. Selecting the type of the stent was based on the availability of the stent and the appropriate stent size. At discharge, the patients continued to take at least 80 mg of aspirin daily for an indefinite period and clopidogrel daily (75 mg) for at least 12 months. The patients were followed up at 1, 6, and 12 months after PCI via clinic visits or telephone interviews. 

The primary end points of this study were 1-year major adverse cardiac events (MACE), comprising death, myocardial infarction, target vessel revascularization, and target lesion revascularization. Target vessel revascularization was defined as any repeat percutaneous intervention or coronary artery bypass grafting of any segment of a previously treated vessel. Target lesion revascularization was defined as a clinically indicated percutaneous or surgical revascularization of an index lesion during the follow-up. The secondary end point was stent thrombosis, defined according to the Academic Research Consortium.^12^

The data are presented as mean ± standard deviation (SD) for the continuous variables and frequency (%) for the categorical variables. The Pearson chi-square or the Fisher exact test was utilized to compare the categorical variables, and the Student *t*-test or the Mann-Whitney test was employed to compare the continuous variables between the study groups, as required. The Kolmogorov-Smirnov test was applied to test for the normal distribution of the continuous variables. The Kaplan-Meier method was drawn upon to estimate the survival curve. The variables which were associated with both MACE and stent type simultaneously (p value ≤ 0.2) were assumed to be possible confounders. The Cox proportional hazard model was used to assess the adjusted association between the stent type and the follow-up outcome. Adjustment was performed on following variables: renal failure, previous bypass surgery, complete revascularization, and multivessel disease variables. A p value < 0.05 was considered statistically significant. The statistical analyses were conducted using SPSS 15 for Windows.

## Results

A total of 949 patients (1018 lesions) were enrolled in the study. From these patients, 591 patients (630 lesions) received the PROMUS^™^ stent and 358 patients (388 lesions) were treated with the BioMatrix^™^ stent. The baseline patient characteristics are illustrated in Table 1, and the baseline lesion and procedural characteristics are depicted in Table 2. The baseline patient characteristics were similar between the 2 groups, with the exception of the frequencies of hypertension, multivessel disease, and history of bypass grafting, which were significantly higher in the BioMatrix^™^ group (Table 1). A comparison of the lesion and procedure characteristics revealed a significant difference in the distribution pattern of the target vessel between the 2 groups (p value = 0.003): The left anterior descending artery was more common in the PROMUS^™^ group and the left circumflex artery was more common in the BioMatrix^™^ group. The mean stent diameter per lesion and the mean stent inflation pressure per lesion were higher in the EES group. Also, the mean stent length in the EES was significantly longer than that of the BES (Table 2). 

**Figure 1:**
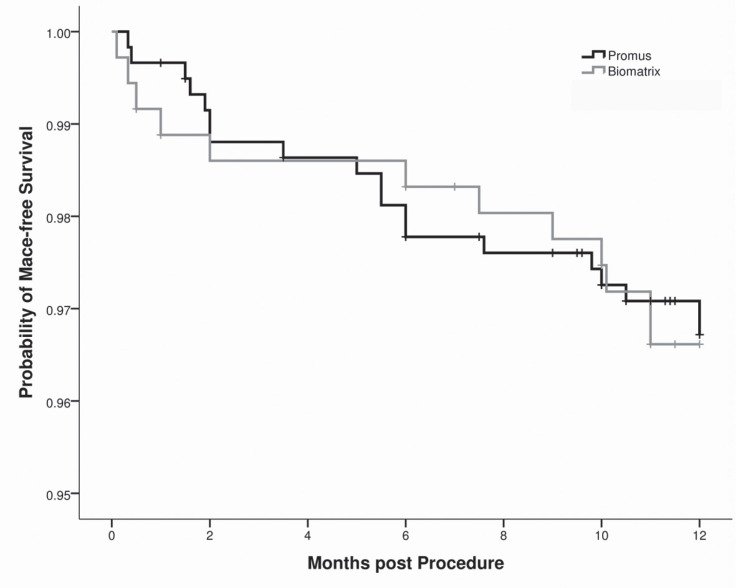
Kaplan-Meier Curve of Major Adverse Cardiac Events-Free Survival

The clinical outcomes at 1 year’s follow-up after PCI are depicted in Table 3. Fourteen (1.5%) patients did not complete their 12 months’ follow-up; the follow-up duration for these patients was between 1 and 9 months, and they were considered as censored cases when performing the Cox proportional hazard model. Of these 14 patients, 9 (1.5%) were in the PROMUS^™^ group and 5 (1.4%) in the BioMatrix^™^ group (p value = 0.876). As is shown in Table 3, the outcomes at 12 months were similar between the groups with respect to death, myocardial infarction, target vessel revascularization, target lesion revascularization, and stent thrombosis. Cumulative clinical MACE at 1 year’s follow-up was 3.2% and 3.4% in the EES and the BES, respectively (p value = 0.925, HR _(EES to BES)_ = 1.035, 95% CI: 0.50 to 2.13). Between the variables regarded as confounders, renal failure, previous bypass surgery, complete revascularization, and multi-vessel disease were significantly correlated with MACE. After adjustment on these variables, there was no statistically significant difference in terms of MACE between the PROMUS^™^ stent and the BioMatrix^™^ stent (p value = 0.598, HR _(EES to BES)_ = 0.817, 95% CI: 0.39 to 1.73).The Kaplan-Meier curve of MACE-free survival is illustrated in Figure 1. There was also no statistically significant difference as regards stent thrombosis between the 2 groups.

**Table 1:** Baseline Patients Characteristics*

	Total (n=949)	EES (n=591)	BES (n=358)	P Value
Age (y)	59.48±10.46	59.24±10.23	59.88±10.83	0.364
Male	629 (66.3)	385 (65.1)	244 (68.2)	0.341
Body mass index (kg/m^2^)	27.81±4.37	27.87±4.54	27.70±4.09	0.570
Abdominal circumference (cm)	100.92±9.98	100.76±10.21	101.17±9.61	0.550
Risk Factors				
Family history of CAD	178 (18.8)	109 (18.5)	69 (19.3)	0.769
Current smoker	220 (23.2)	136 (23.1)	84 (23.5)	0.403
Diabetes	353 (37.2)	212 (35.9)	141 (39.4)	0.286
Hypertension	520 (54.9)	300 (50.8)	220 (61.5)	0.001
Dyslipidemia	634 (67.5)	387 (66.5)	247 (69.2)	0.392
Renal failure	12 (1.3)	4 (0.7)	8 (2.2)	0.067
Previous myocardial infarction	118 (12.4)	72 (12.2)	46 (12.8)	0.763
Previous PCI	98 (10.3)	63 (10.7)	35 (9.8)	0.665
Previous bypass surgery	56 (5.9)	27 (4.6)	29 (8.1)	0.027
Cerebrovascular disease	22 (2.8)	9 (2.1)	13 (3.6)	0.177
Multivessel disease	518 (54.8)	307 (52.3)	211 (58.9)	0.047
Left ventricular ejection fraction (%)	49.99±10.06	50.15±10.04	49.71±10.11	0.537
Clinical status within the recent 2 months				0.266
Stable angina	295 (31.1)	197 (33.3)	98 (27.4)	
Unstable angina	304 (32.0)	180 (30.5)	124 (34.6)	
Non-ST-elevation myocardial infarction	129 (13.6)	79 (13.4)	50 (14.0)	
ST-elevation myocardial infarction	221 (23.2)	135 (22.8)	86 (24.0)	

EES, Everolimus-eluting stent; BES, Biolimus-eluting stent; CAD, Coronary artery disease; PCI, Percutaneous coronary intervention

* Data are presented as mean±SD or n (%).

**Table 2:** Patients and Lesions Characteristics

	Total	EES	BES	P Value
Lesion characteristics	(n=1018)	(n=630)	(n=388)	
Lesion territory				0.003
Left anterior descending	660 (64.8)	434 (68.9)	226 (58.2)	
Left circumflex	179 (17.6)	91 (14.4)	88 (22.7)	
Right coronary	167 (16.4)	98 (15.6)	69 (17.8)	
No. of coronary grafts	12 (1.2)	7 (1.1)	5 (1.3)	
Primary PCI	23 (2.4)	11 (1.9)	12 (3.4)	0.148
Bifurcation lesion	122 (12)	83 (13.2)	39 (10.1)	0.136
Total occlusion	90 (8.8)	55 (8.7)	35 (9)	0.874
Reference vessel diameter	3.13±0.40	3.14±0.37	3.10±0.43	0.141
Pre-procedure diameter stenosis	89.12±7.12	89.15±7.10	89.26±6.70	0.847
Lesion length (mm)	20.72±7.80	21.18±8.06	19.98±7.30	0.018
Stent diameter (mm)	3.02±0.36	3.04±0.32	2.99±0.42	0.046
Stent length (mm)	22.23±5.66	22.62±5.61	21.61±5.69	0.006
Stent inflation pressure (atm)	12.53±2.87	12.68±2.73	12.28±3.07	0. 034
No. of stent per lesion	1.04±0.20	1.04±0.20	1.04±0.20	0.603
Patient characteristics	(n=949)	(n=591)	(n=358)	
No. of stent per patient	1.11±0.33	1.10±0.31	1.13±0.36	0.190
Complete revascularization	462 (49.1)	300 (51.1)	162(45.3)	0.112

Values are mean±SD or n (%).

EES, Everolimus-eluting stent; BES, Biolimus-eluting stent; PCI, Percutaneous coronary intervention.

**Table 3:** One-year major adverse cardiac events (MACE)

	Total (n=949)	EES (n=591)	BES (n=358)	P Value
Total MACE	31 (3.3)	19 (3.2)	12 (3.4)	0.908
Target lesion revascularization	3 (0.3)	2 (0.3)	1 (0.3)	0.999
Target vessel revascularization	8 (0.8)	4 (0.7)	4 (1.1)	0.484
Bypass surgery	3 (0.3)	1 (0.2)	2(0.6)	0.560
PCI	5 (0.5)	3 (0.5)	2 (0.6)	0.999
Myocardial infarction	9 (0.9)	5 (0.8)	4 (1.1)	0.736
Cardiac death	12 (1.3)	9 (1.5)	3 (0.8)	0.551
Noncardiac death	4 (0.4)	2 (0.3)	2 (0.6)	0.635
Stent thrombosis	18 (1.9)	12 (2)	6 (1.7)	0.698
Definite	2 (0.2)	1 (0.2)	1 (0.3)	
Probable	10 (1.1)	6 (1.0)	4 (1.1)	
Possible	6 (0.6)	5 (0.8)	1 (0.3)	

Values are n (%).

EES, Everolimus-eluting stent; BES, Biolimus-eluting stent; PCI, Percutaneous coronary intervention

## Discussion

To our knowledge, this is the first study to compare the 1-year clinical outcome of PCI between patients treated with the BioMatrix^™^ stent (BES) and those treated with the PROMUS^™^ stent (EES). The majority of the studies in this field have focused on first-generation SESs as a reference, against which newer stents are compared.^13^^, ^^14^

The salient finding of the present study was that the 1-year MACE of the PROMUS^™^ stent was similar to that of the BioMatrix^™^ stent among patients who underwent PCI. The result of a recent study by Klauss et al.^14^ (LEADER) showed that, at 2 years’ follow-up, the BES with a biodegradable polymer maintained a similar safety and efficacy profile as the SES with a durable polymer. In our study, the EES and the BES exhibited the same safety and efficacy rates in patients undergoing PCI; however, compared with the study by Klauss et al., the rate of MACE in the current study was low (1-y event rate = 3.3% vs. 11.4%). As is reported, more than three-quarters of the patients in the Klauss et al.^14^ study received 1 or more DESs for an off-label indication, and early and late DES safety was inferior in the patients with off-label characteristics.^15^^, ^^16^ All the patients in our study received clopidogrel at least for 12 months, while in the LEADERS study,^17^ 67.3% of the patients continued clopidogrel/thienopyridine for up to 12 months. The number of stent per patient was not reported in the Klauss et al. study, but this ratio in our study was relatively low in comparison with that in other similar studies.^15^^, ^^16^^, ^^18^ This may explain the low MACE rate in the current study by comparison with the rate reported by the other studies.^13^^, ^^14^^, ^^18^

Previous studies have reported that inflammation and hypersensitivity reaction, induced by durable polymer, may promote local thrombosis.^19^ The BES has a biodegradable polymer, which is released during a 6-9 month period after stent deployment. Nevertheless, in the LEADER study, there was no significant difference between the SES and the BES in regard to stent thrombosis after 9 months to 24 months.^14^ It is also worthy of note that in our study, according to Figure 1, there was no significant difference in the rate of MACE-free survival between the EES and the BES at follow-up periods of 9-12 months. Longer-term follow-up periods are required to assess the effect of biodegradable polymer on reducing stent thrombosis.

In the EXCELLET (Efficacy of XIENCE^™^ / PROMUS^™^ Versus CYPHER^®^ to Reduce Late Loss After Stenting) trial,^13^ there were no statistically significant differences in the rate of clinical events between the EES and the SES; moreover, the EES exhibited similar clinical efficacy compared with the SES at 12 months. Nonetheless, on the basis of the COMPARE trial, which compared efficacy and safety between the EES and the paclitaxel-eluting stent (PES), the 1-year outcome of the EES was more optimal than that of the PES with respect to stent thrombosis, myocardial infarction, target vessel revascularization, and target lesion revascularization.^20^ Also in the SPIRIT IV trial, the EES as compared with the PES conferred a significant reduction in the rates of target lesion revascularization and target vessel revascularization.^18^

In the present study, patients treated with the EES had a significantly higher mean stent length and diameter; however, this difference was not clinically noticeable. 

The present study is a non-randomized single-center investigation and may suffer from a bias inasmuch as it does not consider the probable factors that may influence stent selection. In this study, the patients did not undergo follow-up angiography, which precluded determination of the absolute rate of stent restenosis, and the focus was only on clinically-treated target lesion revascularization. Longer-term follow-up periods are required to assess the efficacy of stents with biodegradable polymer in reducing target lesion revascularization and stent thrombosis rates. This study is retrospective in its nature; accordingly, selection bias cannot be excluded. 

## Conclusion

At 1 year’s follow-up, the BES (BioMatrix^™^ stent) and the EES (PROMUS^™^ stent) showed similar safety and efficacy rates in patients undergoing PCI with a relatively low rate of adverse events. 

## Notes:

***This paper should be cited as:*** *Alidoosti M, Sharifnia V, Kassaian SE, Hajizeinali A, Poorhosseini H, Salarifar M, Nozari Y, Hakki Kazazi E. *One-Year Outcome of Everolimus-Eluting Stents Versus Biolimus-Eluting Stents in Patients Undergoing Percutaneous Coronary Intervention. J Teh Univ Heart Ctr 2016;11(2):62-67.
